# The Effect of Sodium Benzoate on Host Health: Insight into Physiological Indexes and Gut Microbiota

**DOI:** 10.3390/foods12224081

**Published:** 2023-11-10

**Authors:** Nanhai Xiao, Shengyue Ruan, Qiufen Mo, Minjie Zhao, Fengqin Feng

**Affiliations:** 1 College of Biosystems Engineering and Food Science, Zhejiang University, Hangzhou 310058, China; xnh1183126320@163.com (N.X.); rsy19980422@sina.com (S.R.); mqfydws@163.com (Q.M.); minjiezhao@zju.edu.cn (M.Z.); 2 Ningbo Research Institute, Zhejiang University, Ningbo 315100, China; 3 Zhongyuan Institute, Zhejiang University, Zhengzhou 450001, China

**Keywords:** sodium benzoate, gut microbiota, SCFA, glucolipid metabolism, fecal microbiota transplantation

## Abstract

Sodium benzoate (SB) is a common food preservative widely used in the food industry. However, the effects of SB intake on host health at different stages were still unclear. Hence, we investigated the impact of SB with three concentrations (150 mg/kg, 500 mg/kg and 1000 mg/kg) and at three stages (intake for 5-weeks, intake for 10-weeks and removal for 5 weeks) on host health in normal mice. The results showed that SB intake for 5 weeks slightly changed gut microbiota composition, but it significantly increased TG (only 150 mg/kg and 1000 mg/kg) and blood glucose levels (only 500 mg/kg) and promoted the secretion of interleukin (IL)-1β and IL-6 (*p* < 0.01). However, SB intake for 10 weeks mostly maintained normal glucolipid metabolism; although, IL-1β (*p* < 0.01) and IL-6 (*p* < 0.05) levels were also significantly increased and positively regulated the gut microbiota by significantly increasing the relative abundance of *Lactobacillus* and significantly decreasing the relative abundance of *Ileibacterium*. Meanwhile, the safety of SB for host metabolism and gut microbiota was also confirmed via a fecal microbiota transplantation experiment. In addition, we found that SB removal after 10 weeks of intake significantly increased the levels of blood glucose, insulin and HOMA-IR index, which might be attributed to gut microbiota dysbiosis. Mechanistically, these positive effects and negative effects had no close relationship with the concentration of short-chain fatty acids in the gut, which might be associated with metabolites of SB or special bacterial strains. In short, this work provided positive evidence for the safety of SB consumption within the recommended range.

## 1. Introduction

Food additives are used to correct appearance, taste, odor, structure, color and other qualities during food processing [1]. Its applications result from the development of technology and consumer appreciation, because food additives can improve food quality, reduce losses, develop new formulations and extend shelf life [2]. Among these food additives, food preservatives are widely used to prevent the undesirable changes caused by microbial spoilage, oxidation and enzyme activity, including natural substances and synthetic sources, such as sodium dehydroacetate, potassium sorbate and tea polyphenols [2]. Generally, synthetic food preservatives with efficient and economical properties are applied into foods and cosmetic industries. Meanwhile, it exhibits excellent antibacterial activity to multiple microorganisms including bacteria, mold and yeast [3,4]. Obviously, food preservatives bring great sensory enjoyment and commercial convenience for producers and consumers, but potential risks to human health should also be valued [5,6]. Therefore, it is significant to conduct a safety evaluation of food preservatives for health risks.

Sodium benzoate (SB) was the first food preservative approved by the Food and Drug Administration, and it is usually added to carbonated drinks, acidic products, sauces and so on. The Acceptable Daily Intake (ADI) specified by the World Health Organization is 0–5 mg/kg of body weight per day [7]. In China, the ADI of SB is less than 2 g/kg body weight per day, and the maximum usage of SB in beverages is 1 g/kg. However, some recent studies showed that SB had adverse health effects. For example, SB attenuated secondary brain injury by inhibiting neuronal apoptosis and reducing mitochondria-mediated oxidative stress in a rat model [8]. Another study revealed that 500 mg/kg of SB for 8 weeks exacerbated memory loss, anxiety, oxidative stress and increased inflammatory/apoptotic effects in the mouse brain [9]. In some clinical trials, SB could improve cognitive function by increasing estradiol to follicle-stimulating hormone ratios in women with later-phase dementia [10], and it also enhanced treatment adherence in late-life depression patients [11]. In fact, the positive uses of SB in treating mental diseases have been reported, such as depressive disorder, neurodegenerative diseases and autism spectrum disorder [12,13]. However, the defective effects of SB have also been discovered regarding genotoxic effects, reproductive toxicity, hormonal disorders and cell necrosis [14,15,16]. In short, these studies only investigated the effect of SB on different diseases, but its effect on normal individuals remains unclear.

The intestinal microbiota that existed in the gastrointestinal tract has been intensively linked to the physiological functions of the host, and its colonization and evolution show continuous dynamic changes [17]. It has been widely accepted that dietary components change host health or disease progression by regulating the gut microbiota [18,19]. Dietary diversity and food preference make more food additives enter the gastrointestinal tract, which can ultimately influence the gut microbiota. Recently, many studies have stated that food additives affected the progression of diseases by regulating the gut microbiota composition and metabolism pathway, including inflammatory bowel diseases and metabolic syndrome [20,21,22]. As a common food preservative, SB affected the development and growth of *Drosophila melanogaster* by changing the gut microbiota composition and endocrine hormone levels [23]. Moreover, benzoate stimulated insulin secretion and reduced glucose intolerance, but it increased adiposity and impaired glucose tolerance in obese mice [24]. These studies showed that SB could affect host health under different metabolic conditions or experimental individuals. However, the effect of SB intake at different stages on host health and the gut microbiota is still unknown.

Therefore, the aim of this study was to investigate the effect of SB intake with different concentrations and at different stages on body conditions, serum biochemistry and gut microbiota. This work would provide a systematic safety evaluation for SB.

## 2. Materials and Methods

### 2.1. Experimental Reagents

SB was purchased from Wuhan Youji Industry Co. Ltd. (Wuhan, China). Triglycerides (TG), cholesterol (TC), high-density lipoprotein-cholesterol (HDL-C), low-density lipoprotein-cholesterol (LDL-C), glutamic-pyruvic transaminase (GPT), fasting blood glucose, glutamic-oxaloacetic transaminase (GOT) and alkaline phosphatase (AKP) were purchased from Nanjing Jiancheng Bioengineering Institute (Nanjing, China). Insulin, leptin, adiponectin, free fatty acid (FFA), lipopolysaccharide (LPS), tumor necrosis factor-α (TNF-α), interleukin (IL)-6 and IL-1β were purchased from Wuhan Jiyinmei Biotechnology Co., Ltd. (Wuhan, China).

### 2.2. Experimental Methods

#### 2.2.1. Animal Treatment and Experimental Design

C57BL/6 male mice (moderate size, no visible tumor or signs of infection, healthy and disease-free) aged 4–5 weeks (17 ± 2 g) were purchased from Shanghai SLAC Laboratory Animal Co. (Shanghai, China), and all mice were housed in a specific condition with 22 ± 2 °C, 50% humidity and 12 h light-dark cycle. All mice had free access to food and water for the entire experimental period. After one week of acclimation, all mice were randomly divided into four different groups (n = 36 per group) by block randomization via software-generated random number sequence based on body weight:normal control diet (NCD), 150 mg/kg of SB group (BL), 500 mg/kg of SB group (BM) and 1000 mg/kg of SB group (BH). SB was mixed into the feed. The three groups treated with SB were fed with basic feed supplemented with SB for 5 weeks and 10 weeks. Then, basic feed supplemented with SB was removed, and basic feed was given to the three groups for another 5 weeks. The entire experimental period was 15 weeks, and the NCD group was treated with basic feed for 15 weeks. The mice were euthanized every 5 weeks (5 weeks, 10 weeks and 15 weeks, n = 12 mice every time) and samples were collected. The reason for specific doses chosen for SB: maximum usage of SB in beverages is 1 g/kg body weight per day in China. We assumed that an adult (60 kg) drinks beverages (150 g/bottle) three times a day, so the daily intake of SB is 0.45 g. According to the related ratio, the daily intake of mice (20 g) is 0.15 mg. Because the equivalent dose for experimental mice is about ten times that for humans, the daily intake of mice (20 g) is 1.5 mg per day. Daily feed intake of mice is evaluated as 3 g per mouse, so feed should contain 500 mg/kg of SB, and 150 mg/kg of SB was considered as one-third of the maximum dose, and 1000 mg/kg of SB was double.

Fecal microbiota transplantation (FMT) [25]: The mice (healthy and disease-free, aged 4–5 weeks, 17 ± 2 g) fed with a basic diet or 500 mg/kg of SB were considered as the donor mice. The two groups (n = 27 per group) were treated with their own feed for 10 weeks. Then, the feces of two groups during the final 5 weeks were collected and dissolved into 0.9% saline (1:9), and then upper supernatants were obtained to be used as the FMT material after centrifugation at 400× *g* for 10 min at 4 °C. The recipient mice (n = 16 mice per group) were first treated with antibiotic mixtures for 4 weeks to establish pseudo-germ-free mice, including ampicillin (1 g/L), neomycin (1 g/L), metronidazole (1 g/L) and vancomycin (0.5 g/L). Antibiotic mixtures were dissolved into drinking water. Then, recipient mice were administrated with FMT material once per day. Three days later, transplantation treatment was changed once a week, and the duration of FMT treatment was 10 weeks. R group: recipient mice were administrated with FMT material from the NCD group. RB group: recipient mice were administrated with FMT material from the BM group.

The body weight of mice was recorded every week. The mice were fasted overnight and then euthanized under anesthetization with diethyl ether asphyxiation at the end of every experimental period. Fresh fecal samples were collected and stored at −80 °C. Liver, spleen, kidney, epididymal adipose tissue, brown adipose tissue and inguinal subcutaneous adipose tissues were weighted and used to calculate organ index that was expressed as organ weight/body weight. Serum samples were collected and stored at −80 °C until further analysis.

#### 2.2.2. Serum Biochemistry Measurement

Serum biochemistry contained blood lipids, blood glucose, inflammatory factors and organ damage biomarkers. Specifically, blood lipids included TG, TC, LDL-C, HDL-C and FFA. Blood glucose included fasting blood glucose, insulin, leptin and adiponectin. Inflammatory factors contained LPS, TNF-α, IL-6 and IL-1β. GOT, GPT and AKP were considered as liver function markers. The concentrations of these factors were measured using enzyme linked immunosorbent assay (ELISA) kits according to the manufacturer’s protocol. The homeostatic model index of insulin resistance (HOMA-IR) was calculated (HOMA-IR: glucose (mmol/L) × insulin (μmol/mL)/22.5).

#### 2.2.3. Gut Microbiota Analysis

Fresh fecal samples were collected and frozen in liquid nitrogen before storage at −80 °C. Gut microbiota analysis was performed by Shanghai Meiji Biomedical Technology Co., Ltd. (Shanghai, China). Total bacterial DNA was extracted according to the instructions of E.Z.N.A.^®^ soil DNA kit (Omega Bio-tek, Norcross, GA, USA), and the V3-V4 region of 16S rRNA gene was amplified via PCR using the primers 338F (5′-ACTCCTACGGGAGGCAGCAG-3′) and 806R (5′-GGACTACHVGGGTWTCTAAT-3′). The amplified products were performed via the Illumina Miseq platform (Illumina, San Diego, CA, USA) after quality evaluation and quantification. In detail, the resulting sequencing reads (Supplemental Material) were demultiplexed and quality filtered using the following procedures: (i) reads that had more than three consecutive low-quality base calls were discarded using Cutadapt v1.9.1 and were assigned to respective samples according to the unique barcodes, (ii) adaptors and barcodes were trimmed and reads with average quality below Q20 were removed, and (iii) chimeras were dumped using the Userch algorithm (Mo et al., 2021). Then, the sequences with similarity higher than 97% were clustered using UPARSE 11.0 for taxonomic classification, and operational taxon units (OTU) were generated using Silva 138. Venn diagrams, α-diversity (ACE, Shannon index and Simpson index) and β-diversity (based on the unweighted Unifrac distance) were performed. A heatmap was generated on the relative abundance of the top 10% dominant genera using R 2.15.3. Microbiome data have been analyzed by the Kruskal–Wallis H test. The linear discriminant analysis (LDA) effect size (LEfSe) was used to distinguish gut microbiota biomarkers and their corresponding LDA score (LDA > 3.5) were listed.

#### 2.2.4. Short-Chain Fatty Acids Analysis

Short-chain fatty acids (SCFAs) were measured by gas chromatography according to the previous procedure [26]. Briefly, 50 mg of feces was dissolved into ultrapure water. The pH of the fecal suspension was adjusted 2–3 by adding 5 M HCl and the supernatant was obtained after centrifugation. 2-ethylbutyric acid was used as the internal standard. Other information of gas chromatographic analysis was consistent with previous procedure. In detail, a chromatographic column (30 m, 0.53 mm, 0.50 μm) with a free fatty acid phase (DB-FFAP 125–3237, J&W Scientific, Agilent Technologies Inc., Santa Clara, CA, USA) was used for chromatographic analysis. Nitrogen was the carrier at a flow rate of 15 mL/min. The initial oven temperature was set at 100 °C and maintained for 30 s, increased to 180 °C at a rate of 8 °C/min and finally, for 60 s, then raised to 200 °C at 20 °C/min and continued for 15 min. The flame ionization detector and injection port were kept at 240 °C and 200 °C, respectively. The flow rates of hydrogen, nitrogen and air were 30, 20 and 300 mL/min, respectively. The injected volume of each sample for GC analysis was 1 μL, and each analysis had a run period of 27.5 min.

#### 2.2.5. Statistical Analysis

The data were expressed as mean ± SD using GraphPad Prism 6.0 software. Statistical analysis to determine significant differences between groups was performed by SPSS software (version 20). Statistical differences between more than two groups were measured using one-way analysis of variance (ANOVA) with Tukey’s multiple comparison posttests. *p* < 0.05 was considered significant (* *p* < 0.05, ** *p* < 0.01, *** *p* < 0.001).

## 3. Results

### 3.1. SB Intake for 5 Weeks Caused Glucolipid Metabolism Disorder and Promoted Inflammatory Cytokines Secretion

**Figure 1:**
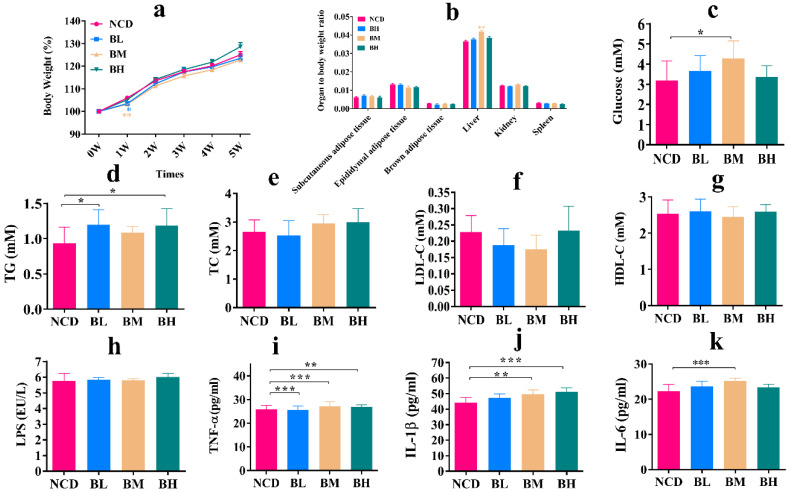
SB intake for 5 weeks (5W) caused glucolipid metabolism disorder and promoted inflammatory cytokines secretion. (**a**) Body weight change for each group (n = 18~35 mice per group). (**b**) Organ to body weight ratio for each group (n = 9~29 mice per group). (**c**) Fasting blood glucose for each group (n = 10 mice per group). (**d**) TG concentration in the serum for each group (n = 8~9 mice per group). (**e**) TC concentration in the serum for each group (n = 8~9 mice per group). (**f**) LDL-C concentration in the serum for each group (n = 9 mice per group). (**g**) HDL-C concentration in the serum for each group (n = 9 mice per group). (**h**) LPS concentration in the serum for each group (n = 8 mice per group). (**i**) TNF-α concentration in the serum for each group (n = 8 mice per group). (**j**) IL-1β concentration in the serum for each group n = 8 mice per group). (**k**) IL-6 concentration in the serum for each group (n = 8 mice per group). Data are presented as mean ± SD. * *p* < 0.05, ** *p* < 0.01, *** *p* < 0.001 versus NCD group. Normal control diet (NCD), 150 mg/kg of SB group (BL), 500 mg/kg of SB group (BM), 1000 mg/kg of SB group (BH).

To investigate the effect of SB intake for 5 weeks on body growth and health condition, weight gain, organ index and serum biochemistry were first estimated. As shown in Figure 1, during the short experimental period, the body weights of the four groups showed a gradually increasing trend with increasing time, and body weights of the BL group and BM group were significantly higher than the NCD group in the first week (Figure 1a). Meanwhile, there was no significant difference between the three groups with SB and the NCD group for the organ index; although, the liver/body ratio was significant between the BM group and NCD group (Figure 1b). These results indicated that SB intake for 5 weeks could maintain normal body growth and organ index. The levels of blood glucose and blood lipids reflected a metabolism change, and inflammatory factors such as cytokines and LPS deduced immune system status. To further explore whether SB intake influenced immune homeostasis and metabolic processes, glucolipid metabolism parameters and inflammatory cytokines were measured. The results pointed out that SB intake with various concentrations for 5 weeks prominently increased the level of fasting blood glucose (only BM group) (Figure 1c), TG (only BL and BH group) (Figure 1d), TNF-α (only BM and BH group) (Figure 1i), IL-1β (only BM and BH group) (Figure 1j) and IL-6 (only BM group) (Figure 1k) compared with the NCD group, but it had no obvious effects on LPS, TC, LDL-C and HDL-C concentrations, which demonstrates that SB intake for 5 weeks caused partial glucolipid metabolism disorder and promoted inflammatory cytokines secretion.

### 3.2. SB Intake for 5 Weeks Slightly Changed Gut Microbiota Composition

**Figure 2:**
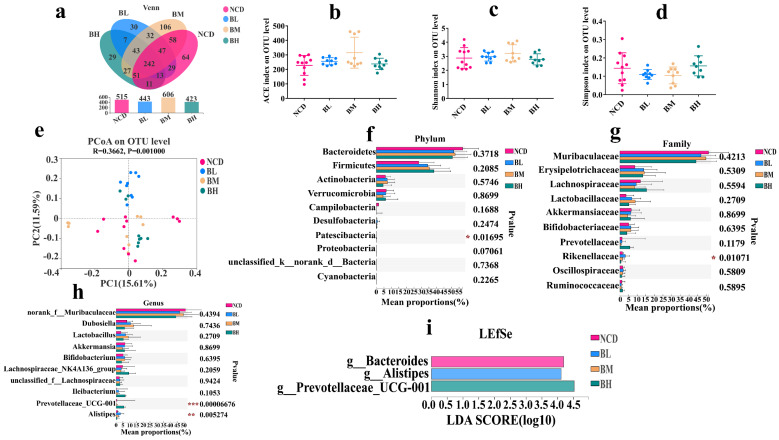
SB intake for 5 weeks slightly changed gut microbiota composition. (**a**) Venn diagrams of the OTU illustrated overlap among groups. (**b**) ACE index on OTU level. (**c**) Shannon index on OTU level. (**d**) Simpson index on OTU level. (**e**) PCoA analysis based on the unweighted Unifrac distance. (**f**) Relative abundance (%) at the phylum level of each group. (**g**) Relative abundance (%) at the family level of each group. (**h**) Relative abundance (%) at the genus level of each group. (**i**) LEfSe analysis of gut microbiome of each group. Data are presented as mean ± SD. * *p* < 0.05, ** *p* < 0.01, *** *p* < 0.001 versus NCD group. n = 9~11 mice per group.

To explore the effect of SB intake for 5 weeks on gut microbiota, 16S rDNA sequencing was performed. The α diversity is the indicator that represents the richness and species diversity of gut microbiota including OTU number, ACE index, Shannon index and Simpson index. As shown in Figure 2, no significant effect was observed on the OTU number, ACE index, Shannon index or Simpson index (Figure 2a–d) compared with the NCD group, which implied that SB intake for 5 weeks had no obvious impact on α diversity of the gut microbiota. Then, the β diversity reveals the similarity of the gut microbiota composition based on the distances between points. The result showed that the points of every group were scattered, and the distances between different groups were less obvious in the PCoA of unweighted Unifrac (ANOSIM R = 0.3662, *p* = 0.001) (Figure 2e), which deduced that the gut microbiota composition of the four groups might be similar. Therefore, we further analyzed gut microbiota compositions. The results showed that *Bacteroidetes*, *Firmicutes*, *Actinobacteria* and *Verrucomicrobia* were the dominant bacteria in the four groups, and there were no significant differences in the relative abundance between each group at the phylum level (Figure 2f). However, the abundance of *Rikenellaceae* was dramatically changed after SB intervention at the family level (Figure 2g). At the genus level, we found that the abundances of *Lactobacillus* and *Bifidobacterium* increased; although, it was statistically insignificant. The abundances of *Prevotellaceae UCG-001* and *Alistipes* were noteworthily up-regulated, and others were insignificant compared to the NCD group (Figure 2h). To identify the biomarkers of the gut microbiota, an LEfSe analysis (LDA > 3.5) was employed. The LEfSe results revealed that the NCD group had a unique *Bacteroides* at the genus level, and the BL group and BH group had *Alistipes* and *Prevotellaceae UCG-001* (Figure 2i). In a word, SB intake for 5 weeks slightly changed the gut microbiota composition, and it showed no dose-dependent manner.

### 3.3. SB Intake for 10 Weeks Significantly Affected Secretion of Inflammatory Cytokines

**Figure 3:**
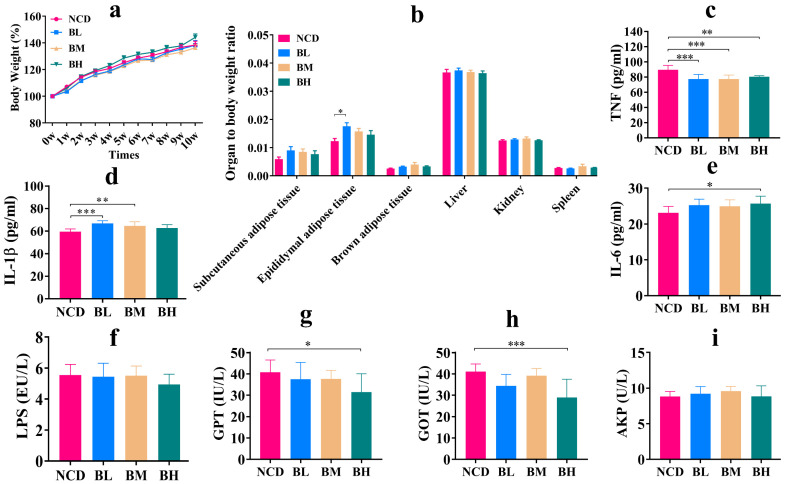
SB intake for 10 weeks (10W) significantly affected secretion of inflammatory cytokines. (**a**) Body weight change for each group (n = 9~24 mice per group). (**b**) Organ to body weight ratio for each group (n = 9~12 mice per group). (**c**) TNF-α concentration in the serum for each group (n = 7~8 mice per group). (**d**) IL-1β concentration in the serum for each group (n = 8 mice per group). (**e**) IL-6 concentration in the serum for each group (n = 8 mice per group). (**f**) LPS concentration in the serum for each group (n = 6~8 mice per group). (**g**) GPT concentration in the serum for each group (n = 7~8 mice per group). (**h**) GOT concentration in the serum for each group (n = 5~8 mice per group). (**i**) AKP concentration in the serum for each group (n = 9 mice per group). Data are presented as mean ± SD. * *p* < 0.05, ** *p* < 0.01, *** *p* < 0.001 versus NCD group.

We have previously investigated the effect of SB intake for 5 weeks on the health condition and gut microbiota and found that short-term SB intake partially caused glucolipid metabolism disorder and promoted inflammatory cytokines secretion. However, the effect of SB intake over an extended period was still unclear. Hence, the normal mice were administrated with SB for 10 weeks to investigate its influence on body changes, immune condition and gut microbiota. As shown in Figure 3, SB intervention for 10 weeks showed no noticeable effect on body weight and organ/body index compared with the NCD group; although, 150 mg/kg of SB markedly increased the epididymal adipose tissue/body ratio (Figure 3a,b), which indicated that SB intake with different concentrations for 10 weeks showed no obvious damage to body conditions. Then, to evaluate the degree of immune system activation, we measured the concentration of inflammatory factors in the serum. The result proved that SB intervention significantly reduced TNF-α concentration, and significantly promoted IL-1β and IL-6 secretions compared with the NCD group, but it had no obvious impact on the LPS concentration (Figure 3c–f). These results revealed that SB intake prominently affected the secretion of inflammatory cytokines. To further estimate whether the SB intake for 10 weeks caused organ function imbalance, we analyzed the levels of GOT, GPT and AKP, because GOT, GPT and AKP were known as liver function markers. We found that SB intervention for 10 weeks basically showed no evident influence on biomarker concentration compared with the NCD group; although, 1000 mg/kg of SB distinctly inhibited GOT and GPT secretions (Figure 3g–i), which demonstrated that SB was a relatively safe food additive without obvious risk of liver damage. In short, the SB intake for 10 weeks had no apparent effect on body conditions and organic damage, but significantly affected the secretion of inflammatory cytokines.

### 3.4. SB Intake for 10 Weeks Mostly Maintained Normal Glucolipid Metabolism

**Figure 4:**
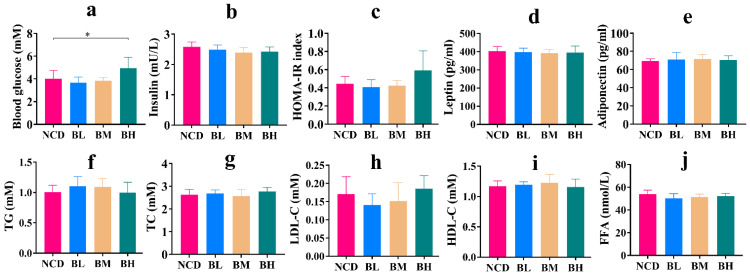
SB intake for 10 weeks mostly maintained normal glucolipid metabolism. (**a**) Fasting blood glucose for each group (n = 7~9 mice per group). (**b**) Insulin concentration in the serum for each group (n = 8 mice per group). (**c**) HOMA-IR for each group (n = 7~8 mice per group). (**d**) Leptin concentration in the serum for each group (n = 8 mice per group). (**e**) Adiponectin concentration in the serum for each group (n = 8 mice per group). (**f**) TG concentration in the serum for each group (n = 9 mice per group). (**g**) TC concentration in the serum for each group (n = 9 mice per group). (**h**) LDL-C concentration in the serum for each group (n = 8~9 mice per group). (**i**) HDL-C concentration in the serum for each group (n = 8~9 mice per group). (**j**) FFA concentration in the serum for each group (n = 8 mice per group). Data are presented as mean ± SD. * *p* < 0.05 versus NCD group.

Normal glucolipid metabolism is of importance for physiological homeostasis, because glucose metabolism interacts with lipid metabolism, which easily results in metabolic syndromes such as obesity and diabetes. So, to investigate whether SB intake for 10 weeks caused glucolipid metabolism disorder, some hormones and compounds related to glucolipid metabolism in the serum were measured. As shown in Figure 4, the results revealed that there was no remarkable difference in the concentration of TG, TC, LDL-C, HDL-C and FFA between the three SB groups and the NCD group (Figure 4f–j). However, the level of fasting blood glucose in the BH group was dramatically higher than the NCD group, but the insulin level and HOMA-IR index were insignificant. Meanwhile, the BL group and BM group were also insignificant compared to the NCD group for the fasting glucose level, insulin level and HOMA-IR index (Figure 4a–c), which deduced that a high concentration of SB might cause blood glucose metabolism disorder. Finally, hormone results exhibited that a long-term intake of SB had no obvious effect on leptin and adiponectin levels compared with the NCD group (Figure 4d,e). In brief, the SB intake for 10 weeks mostly maintained normal glucolipid metabolism, but a high concentration significantly increased the concentration of fasting blood glucose.

### 3.5. SB Intake for 10 Weeks Positively Regulated Gut Microbiota

**Figure 5:**
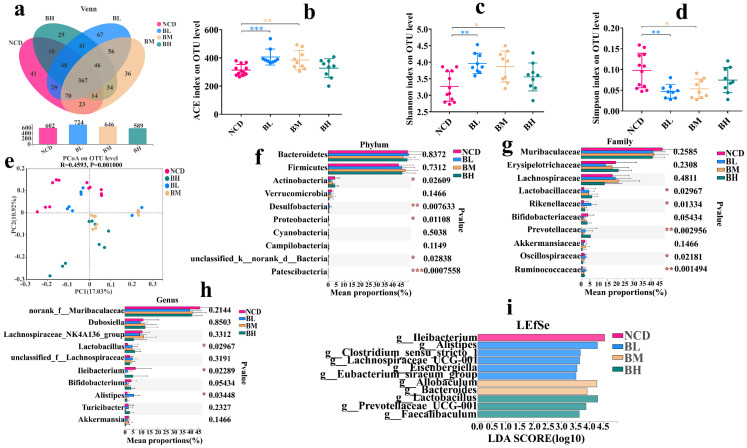
SB intake for 10 weeks positively regulated gut microbiota. (**a**) Venn diagrams of the OTU illustrated overlap among groups. (**b**) ACE index on OTU level. (**c**) Shannon index on OTU level. (**d**) Simpson index on OTU level. (**e**) PCoA analysis based on the unweighted Unifrac distance. (**f**) Relative abundance (%) at the phylum level of each group. (**g**) Relative abundance (%) at the family level of each group. (**h**) Relative abundance (%) at the genus level of each group. (**i**) LEfSe analysis of gut microbiome of each group. Data are presented as mean ± SD. * *p* < 0.05, ** *p* < 0.01, *** *p* < 0.001 versus NCD group. n = 9~12 mice per group.

We confirmed that SB intake for 5 weeks slightly changed the gut microbiota composition, but the effect of SB intake over an extended period on the gut microbiota composition remained unknown. Therefore, we analyzed the change in the gut microbiota composition after SB administration for 10 weeks. As shown in Figure 5, there was no significant difference in the OTU number among the four groups (Figure 5a). However, we found that the ACE index and Shannon index of the BL group and BM group were signally higher than the NCD group, and the Simpson index was significantly lower, but the BH group had no similar phenomenon, which implied that the SB intakes with 150 mg/kg and 500 mg/kg but not 1000 mg/kg obviously changed the α diversity of the gut microbiota (Figure 5b–d). Then, the β diversity result showed that the points of every group were dispersive, and the distances between the different groups were obvious (Figure 5e), which deduced that the gut microbiota compositions of the four groups were distinctly different. Therefore, we further analyzed their gut microbiota compositions. The results showed that the main bacteria were *Bacteroidetes*, *Firmicutes*, *Actinobacteria* and *Verrucomicrobia* in the four groups. At the phylum level, the abundance of *Actinobacteria*, *Desulfobacteria*, *Proteobacteria* and *Patescibacteria* were dramatically changed (Figure 5f). At the family level, the abundances of *Lactobacillaceae*, *Rikenellaceae*, *Prevotellaceae* and *Ruminococcaceae* were significantly up-regulated after the long-term SB intervention (Figure 5g). Importantly, we found that the abundance of *Lactobacillus* was significantly increased, and the abundance of *Ileibacterium* was significantly decreased at the genus level (Figure 5h). An LEfSe analysis revealed that the NCD group had unique *Ileibacterium* at the genus level. The BL group had *Alistipes*, *Clostridium_sensu_stricto_1*, *Lachnospiraceae_UGG_001*, *Eisenbergiella* and *Eubacterium_siraeum_group*. The BM group had *Allobaculum* and *Bacteroides*. The BH group had *Lactobacillus*, *Prevotellaceae UGG_001* and *Faecalibaculum* (Figure 5i). In a word, SB intake for 10 weeks positively regulated the gut microbiota composition.

### 3.6. Fecal Microbiota Transplantation Experiment Confirmed the Safety of SB

**Figure 6:**
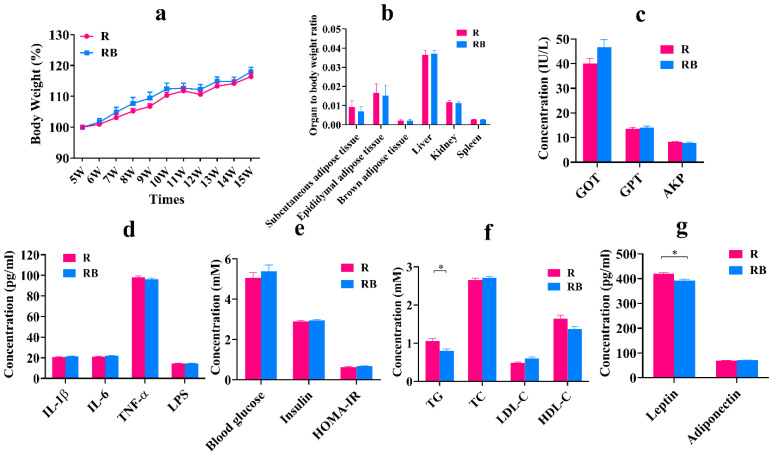
Fecal microbiota transplantation experiment confirmed the safety of sodium benzoate. (**a**) Body weight change for each group (n = 9~15 mice per group). (**b**) Organ to body weight ratio for each group (n = 10~16 mice per group). (**c**) The concentration of GOT, GPT and AKP in the serum for each group (n = 10~16 mice per group). (**d**) The concentration of IL-1β, IL-6, TNF-α and LPS in the serum for each group (n = 10~16 mice per group). (**e**) The concentration of blood glucose, insulin and HOMA-IR in the serum for each group (n = 10~16 mice per group). (**f**) The concentration of TG, TC, LDL-C and HDL-C in the serum for each group (n = 10~16 mice per group). (**g**) The concentration of leptin and adiponectin in the serum for each group (n = 10~16 mice per group). Data are presented as mean ± SD. * *p* < 0.05 versus R group. R group: recipient mice were administrated with FMT material from NCD group. RB group: recipient mice were administrated with FMT material from BM group.

To explain whether the gut microbiota played a critical role in the safety evaluation of SB, fecal microbiota materials derived from the BM group were fed to the receipt mice. As shown in Figure 6, the results testified that fecal microbiota materials derived from the BM group first had no prominent influence on body condition, organ index and organ damage biomarkers (Figure 6a–c). Then, from the view of inflammatory factors, LPS, TNF-α, IL-1β and IL-6 concentrations of the RB group were insignificant compared with the R group (Figure 6d). Then, we also explored the changes in glucolipid metabolism in the serum after fecal microbiota transplantation and found that most indexes related to glucolipid metabolism showed no significant differences, but the TG level and leptin level were only markedly decreased compared with the R group (Figure 6e–g). However, in general, the FMT experiment confirmed that the gut microbiota played an essential role in the safety evaluation of SB, which could not cause obvious abnormal changes in body conditions, inflammatory factors and glucolipid metabolism.

### 3.7. Fecal Microbiota Transplantation Significantly Changed Gut Microbiota Composition of Receipt Mice

**Figure 7:**
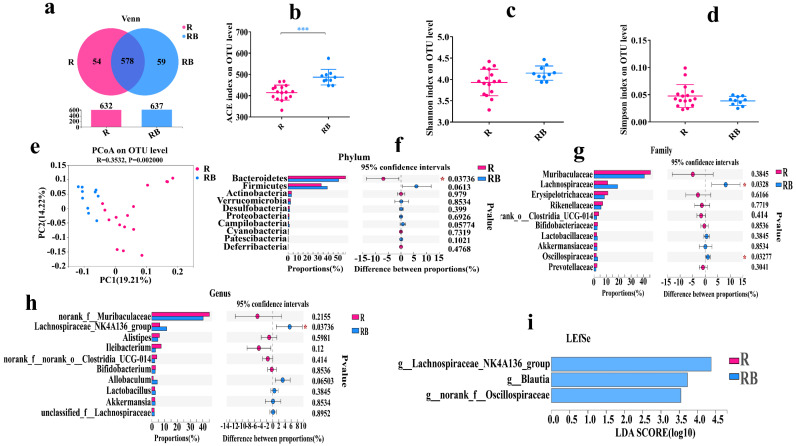
Fecal microbiota transplantation for 10 weeks significantly changed gut microbiota composition of receipt mice. (**a**) Venn diagrams of the OTU illustrated overlap among groups. (**b**) ACE index on OTU level. (**c**) Shannon index on OTU level. (**d**) Simpson index on OTU level. (**e**) PCoA analysis based on the unweighted Unifrac distance. (**f**) Relative abundance (%) at the phylum level of each group. (**g**) Relative abundance (%) at the family level of each group. (**h**) Relative abundance (%) at the genus level of each group. (**i**) LEfSe analysis of gut microbiome of each group. Data are presented as mean ± SD. * *p* < 0.05, *** *p* < 0.001 versus R group. n = 10~16 mice per group. R group: recipient mice were administrated with FMT material from NCD group. RB group: recipient mice were administrated with FMT material from BM group.

We have found that SB intake for 10 weeks positively regulated the gut microbiota composition, especially significantly increasing *Lactobacillus* abundance and significantly decreasing *Ileibacterium* abundance, but whether this effect was gut-microbiota dependent was unclear. Therefore, we further analyzed the gut microbiota composition of the receipt mice. As shown in Figure 7, the results showed that the ACE indexes of the RB group were markedly higher than those of the R group, but the OTU numbers, Shannon indexes and Simpson indexes were insignificant between the two groups, which demonstrated that fecal microbiota materials derived from the BM group notably enhanced the richness but not diversity of the gut microbiota in the receipt mice (Figure 7a–d). Then, from the result of β diversity, we found that the points of the two groups exhibited obvious distances, and it stated that their gut microbiota compositions were different (Figure 7e). Therefore, we further examined the gut microbiota compositions. The results showed that the main bacteria were *Bacteroidetes*, *Firmicutes*, *Actinobacteria* and *Verrucomicrobia* at the phylum level in the two groups, in which the abundance of *Bacteroidota* was significantly reduced, and the abundance of *Firmicutes* was enhanced in the RB group after FMT (Figure 7f). At the family level, the abundances of *Lachnospiraceae* and *Oscillospiraceae* were significantly improved (Figure 7g). At the genus level, the abundances of *Lachnospiraceae_NK4A136_group* and *Lactobacillus* were increased, and the abundance of *Ileibacterium* was decreased (Figure 7h). The LEfSe result also confirmed that the RB group had unique *Lachnospiraceae* and *Oscillospiraceae* (Figure 7i). The changed trends of these results were similar to previous results, which deduced that the gut microbiota was a necessary factor for SB safety.

### 3.8. The Removal of SB Had No Significant Effect on Body Conditions and Systemic Inflammation

**Figure 8:**
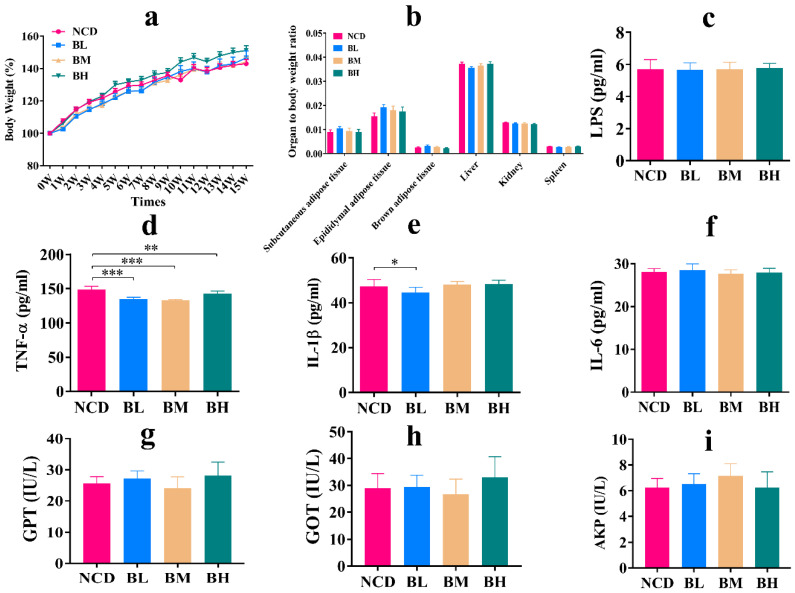
The removal of SB had no significant effect on body conditions and systemic inflammation. (**a**) Body weight change for each group (n = 9~12 mice per group). (**b**) Organ to body weight ratio for each group (n = 9~12 mice per group). (**c**) LPS concentration in the serum for each group (n = 6~8 mice per group). (**d**) TNF-α concentration in the serum for each group (n = 7~8 mice per group). (**e**) IL-1β concentration in the serum for each group (n = 8 mice per group). (**f**) IL-6 concentration in the serum for each group (n = 8 mice per group). (**g**) GPT concentration in the serum for each group (n = 8 mice per group). (**h**) GOT concentration in the serum for each group (n = 7~8 mice per group). (**i**) AKP concentration in the serum for each group (n = 8 mice per group). Data are presented as mean ± SD. * *p* < 0.05, ** *p* < 0.01, *** *p* < 0.001 versus NCD group.

The effects of SB intake for 5 weeks and 10 weeks on body conditions, serum biochemistry and gut microbiota were already investigated, but the influence of SB removal for a normal individual was still unclear. Therefore, we measured the effect of SB removal on body conditions and systemic inflammation. As shown in Figure 8, the result indicated that the removal of SB with three concentrations for an additional 5 weeks showed no noticeable effect on body weight and organ/body index compared with the NCD group (Figure 8a,b). Then, inflammatory factors in the serum were analyzed. We found that the concentrations of LPS, IL-1β and IL-6 were mostly insignificant compared with the NCD group, but the TNF-α level was notably inhibited after SB removal (Figure 8c–f), which implied that the removal of SB with three concentrations for an additional 5 weeks caused no systemic inflammation. Finally, liver function markers were measured. The result showed that there were no significant differences in GPT, GOT and AKP concentrations (Figure 8g–i), which meant that SB removal was without obvious organ damage. In brief, SB removal had no significant effects on body conditions and systemic inflammation.

### 3.9. The Removal of SB Caused Glucose Metabolism Disorder

**Figure 9:**
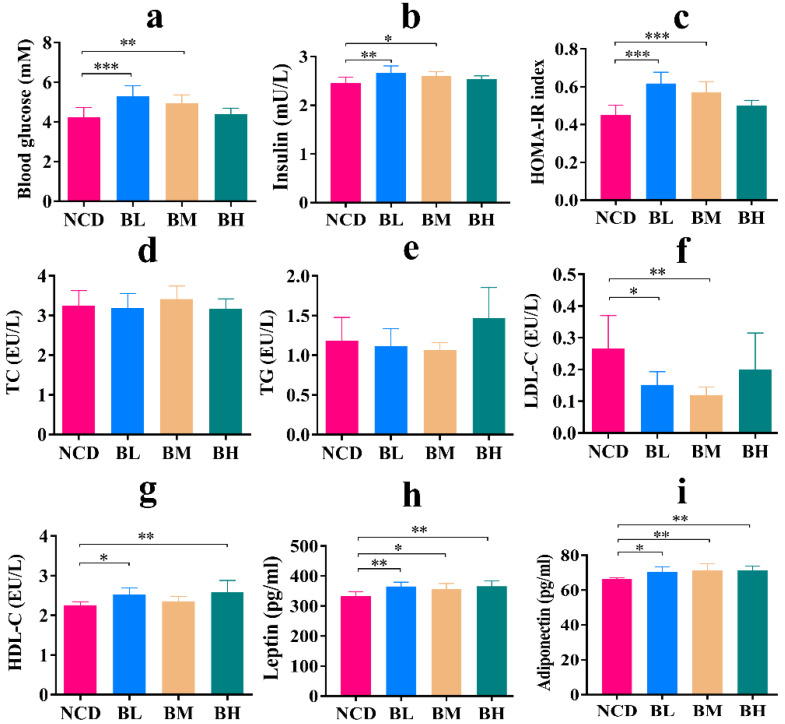
The removal of SB caused glucose metabolism disorder. (**a**) Fasting blood glucose for each group (n = 8~9 mice per group). (**b**) Insulin concentration in the serum for each group (n = 8 mice per group). (**c**) HOMA-IR for each group (n = 7~8 mice per group). (**d**) TC concentration in the serum for each group (n = 9 mice per group). (**e**) TG concentration in the serum for each group (n = 9 mice per group). (**f**) LDL-C concentration in the serum for each group (n = 7~9 mice per group). (**g**) HDL-C concentration in the serum for each group (n = 8~9 mice per group). (**h**) Leptin concentration in the serum for each group (n = 8 mice per group). (**i**) Adiponectin concentration in the serum for each group (n = 8 mice per group). Data are presented as mean ± SD. * *p* < 0.05, ** *p* < 0.01, *** *p* < 0.001 versus NCD group.

Glucolipid metabolism is closely associated with host health, although SB intake for 10 weeks mostly maintained normal glucolipid metabolism, and whether this effect would continue after SB removal is unknown. As shown in Figure 9, the results revealed that TG and TC levels of the three groups showed no prominent differences compared with the NCD group, but LDL-C levels of the BL group and BM group were markedly decreased, and HDL-C levels of the BL group and BH group were dramatically improved (Figure 9d–g). It meant that SB removal might be beneficial to cholesterol metabolism. However, fasting blood glucose, insulin levels and HOMA-IR index of the BL group and BM group were significantly increased (Figure 9a–c). Meanwhile, the leptin level and adiponectin level of the three groups were also significantly increased compared with the NCD group (Figure 9h–i), which declared that SB removal damaged the glucose metabolism of normal mice and even developed insulin resistance. In a word, the removal of SB caused glucose metabolism disorder but not lipid metabolism.

### 3.10. The Removal of SB Changed Gut Microbiota Composition

**Figure 10:**
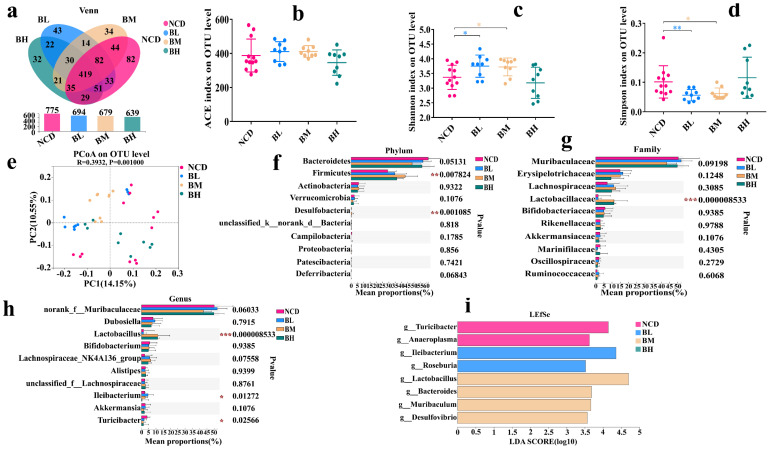
The removal of SB changed gut microbiota composition. (**a**) Venn diagrams of the OTU illustrated overlap among groups. (**b**) ACE index on OTU level. (**c**) Shannon index on OTU level. (**d**) Simpson index on OTU level. (**e**) PCoA analysis based on the unweighted Unifrac distance. (**f**) Relative abundance (%) at the phylum level of each group. (**g**) Relative abundance (%) at the family level of each group. (**h**) Relative abundance (%) at the genus level of each group. (**i**) LEfSe analysis of gut microbiome of each group. Data are presented as mean ± SD. * *p* < 0.05, ** *p* < 0.01, *** *p* < 0.001 versus NCD group. n = 9~12 mice per group.

Based on previous results, we found that SB intake for 10 weeks positively regulated the gut microbiota composition. Nevertheless, the effect of SB removal on the gut microbiota was unclear. As shown in Figure 10, the result showed that the Shannon indexes of the BL group and BM group were markedly higher than the NCD group, and the Simpson indexes of the two groups were also significantly lower, but OTU numbers and ACE indexes were insignificant between each group (Figure 10a–d), which demonstrated that SB removal notably enhanced the diversity of the gut microbiota but not species richness. Then, the β diversity result showed that the points of each group were scattered, And the distances between different groups were less obvious (Figure 10e). Finally, the gut microbiota composition was examined and the result exhibited that *Bacteroidetes*, *Firmicutes*, *Actinobacteria* and *Verrucomicrobia* were the main bacteria at the phylum level in the four groups, in which the abundance of *Firmicutes* was notably enhanced compared with the NCD group (Figure 10f). At the family level, the abundance of *Lactobacillaceae* was significantly increased in the BM group and BH group (Figure 10g). At the genus level, the abundances of *Lactobacillus* in the BM group and BH group and the abundances of *Ileibacterium* in the BL group were notably increased, and the abundance of *Turicibacter* was dramatically decreased compared with the NCD group (Figure 10h). The LEfSe result also confirmed that the four groups had unique biomarkers of gut microbiota (Figure 10i). These changes were inconsistent with previous results of the long-term intake of SB.

### 3.11. The Intake and Removal of SB Hardly Changed the SCFA Production

**Figure 11:**
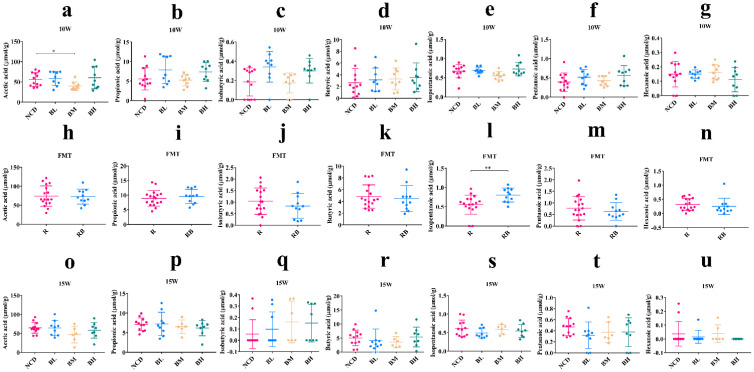
The intake and removal of SB hardly changed the SCFA production. The effect of SB intake for 10 weeks on the SCFA production, acetic acid (**a**), propionic acid (**b**), isobutyric acid (**c**), butyric acid (**d**), isopentanoic acid (**e**), pentanoic acid (**f**) and hexanoic acid (**g**). The effect of FMT on the SCFA production, acetic acid (**h**), propionic acid (**i**), isobutyric acid (**j**), butyric acid (**k**), isopentanoic acid (**l**), pentanoic acid (**m**) and hexanoic acid (**n**). The effect of SB removal on the SCFA production, acetic acid (**o**), propionic acid (**p**), isobutyric acid (**q**), butyric acid (**r**), isopentanoic acid (**s**), pentanoic acid (**t**) and hexanoic acid (**u**). * *p* < 0.05, ** *p* < 0.01 versus NCD group or R group. n = 9~17 mice per group. R group: recipient mice were administrated with FMT material from NCD group. RB group: recipient mice were administrated with FMT material from BM group.

Previous results showed that the intake or removal of SB could significantly increase the abundance of *Lactobacillus* compared with the NCD group. *Lactobacillus* as a kind of SCFA-producing bacteria has been widely reported. So, to evaluate the effect of SB intake and SB removal on intestinal metabolites, the SCFA concentration was measured. As shown in Figure 11, there was no significant effect on the SCFA production after SB intake with three concentrations for 10 weeks, including acetic acid, propionic acid, isobutyric acid, butyric acid, isopentanoic acid, pentanoic acid and hexanoic acid (Figure 11a–g). The acetic acid level of the BM group was even lower than the NCD group. Similarly, the FMT treatment also showed no marked effect on SCFA production (Figure 11h–n). Interestingly, the isopentanoic acid level was significantly enhanced after FMT, but it was not the main SCFA, and its concentration was low. Moreover, no effect on SCFA production after SB removal was found (Figure 11o–u). These results indicated that the intake and removal of SB hardly changed the SCFA production.

## 4. Discussion

As a widely used food preservative, SB is regarded as a safe food additive [27]. However, recent studies reported the beneficial or harmful effects of SB [8,10,27]. In other words, its use is controversial, and a comprehensive evaluation of SB is of great importance. In this study, we investigated the influence of SB with three concentrations (150 mg/kg, 500 mg/kg and 1000 mg/kg) and at three stages (intake for 5-weeks, intake for 10-weeks and removal for 5 weeks) on host health in normal mice. We found that SB intake for 10 weeks was safe for the normal individual, but short-intake for 5 weeks or removal of SB might cause some adverse effects such as increased blood glucose and IL-1β levels, which provided positive evidence for the safety of SB consumption within the recommended range.

Glucolipid metabolism is the primary source of energy in the body, and its homeostatic balance is essential for the body’s internal and external environments [28]. In this study, we found that although SB intake had no obvious influence on body growth and organ index during the whole experimental period, SB intake for 5 weeks significantly increased fasting blood glucose and TG levels, which might be attributed to the early stress response of normal mice [29]. Certainly, it could also be explained by the fact that benzoate impaired glucose tolerance by regulating insulin secretion and pancreas β cell proliferation [24]. SB is a water-soluble food additive that can be quickly absorbed in the gastrointestinal tract and finally forms hippurate by combining with glycine to promote its excretion of SB. Hippurate as an inhibitor of glucose utilization in the muscle was confirmed, and this could be explained by short-term impaired glucose metabolism [30]. Because of the presence of hippurate, the increased level of fasting blood glucose and TG might be explained. Furthermore, the FMT experiment also supported that most indexes related to glucolipid metabolism showed no significant differences after a long intake of SB, although TG and leptin levels were signally changed. Other preservatives also brought undesirable side-effects and changed the sensory and nutritional properties [31,32]. These results stated that SB intake for 10 weeks was relatively safe on glucolipid metabolism. Interestingly, we found that fasting blood glucose and insulin levels of the BL group and BM group were significantly higher than the NCD group after SB removal, which meant that SB removal was helpful in developing insulin resistance. Meanwhile, the leptin and adiponectin levels of the three groups were also markedly higher than the NCD group. Generally, leptin and adiponectin are responsible for increasing insulin sensitivity and preventing ectopic lipid accumulation [33]. Therefore, we guessed that SB removal might impair insulin sensitivity of normal mice, which reduced the degree of glucose consumption and produced the compensatory increase in hormones, ultimately resulting in the increase in the blood glucose level and related hormone levels. However, the detailed gene expression of insulin sensitivity needs to be further investigated. On the contrary, we found that SB removal dramatically decreased the LDL-C level and notably enhanced the HDL-C level in the serum. Serum HDL-C is known as a vascular scavenger and LDL-C is considered as a vascular killer [34,35]. These results inferred that SB removal could regulate lipid metabolism and reduce LDL accumulation. In brief, we concluded that SB intake for 10 weeks had no obvious side-effect on glucolipid metabolism in normal mice, but an apparent glucose metabolism disorder was induced after SB removal.

Low-grade inflammation is a normal physiological reaction to maintain body homeostasis and resist external activation, but an uncontrolled inflammation reaction causes the systemic disorder. In this study, we found that IL-1β and IL-6 levels in the serum were dramatically increased after SB intake for 5 weeks and 10 weeks, which may be attributed to the fact that adipose tissue contributed about 30% of circulating IL-6 [36]. The results for the adipose tissue/body weight ratio could support this inference. Meanwhile, adipocytes also released TNF-α and IL-1β. Hence, we supposed that SB intake might produce slight adipose tissue inflammation, causing the increase in these cytokines. Moreover, the reason for the reduced TNF-α level after SB intake or SB removal was unclear and it needs to be further investigated; although, a recent study also found that three groups fed SB with 125, 250 and 500 mg/kg reduced the serum TNF-α level [37]. A similar phenomenon also confirmed that a high concentration of SB (200, 400, 700 mg/kg) for 30 days produced a significant increase in IL-1β and IL-6 levels in male Wister rats [38]. Interestingly, SB removal decreased serum IL-1β and IL-6 levels, which suggested that SB removal terminated the intervention of SB or its metabolites on the body’s immune system. It also could be explained by the fact that glycine reduced the expression of pro-inflammatory cytokines, because SB removal maintained the glycine level in the serum by eliminating the production of hippurate metabolized by SB [39]. These results revealed the negative effect of SB on immune homeostasis, and the related mechanisms could be confirmed by metabolome.

Recently, the relationship among host health, dietary components and gut microbiota has been widely studied [40]. Considering their wide use and long-term impact, it is necessary to evaluate the effect of food preservatives on the gut microbiota [17]. For example, glycerol monocaprylate significantly increased the relative abundance of *Lactobacillus* at the low-dose concentration and increased the abundance of SCFA-producers at the high-dose concentration [41]. As a common food preservative, the effect of SB on gut microbiota has been reported. In a *Drosophila melanogaster* larvae model, 2000 ppm of SB depleted the bacteria of *Acetobacteraceae* and *Lactobacillus* in the gut [23], and 15 mg/kg/day of SB for 8 weeks significantly reduced the abundance of *Bifidobacterium* [29]. These latest studies confirmed that SB affected the gut microbiota composition, but the effects of different concentrations and different periods on the gut microbiota were lacking. In this study, we found that SB intake for 5 weeks showed a slight effect on the gut microbiota in normal mice. However, SB intake for 10 weeks significantly enhanced the abundance of *Lactobacillus* and dramatically decreased the abundance of *Ileibacterium*. *Lactobacillus* as a beneficial bacterium has widely been considered, and *Ileibacterium* was regarded as a harmful bacterium [42,43]. The result of the FMT experiment also confirmed similar changes, although it was insignificant. Moreover, we found the abundance of *Lachnospiraceae_NK4A136_group* was sharply increased after FMT. *Lachnospiraceae_NK4A136_group* as a butyrate-producing bacterium could maintain intestinal barrier integrity and protect against obesity [44]. These results showed that SB positively regulated the gut microbiota composition and improved bacteria diversity. Generally, the gut microbiota affected host health via their metabolites such as SCFA. Based on potential SCFA-producing property of *Lactobacillus* and *Lachnospiraceae_NK4A136_group*, we measured the concentration of SCFA in the gut. The result showed that the SCFA levels of three groups were insignificant compared with the NCD group. FMT experiment also demonstrated a similar result. These data indicated that SCFA might not be effective gut metabolites. However, at the genus level, SB removal significantly increased the abundances of *Lactobacillaceae* and *Ileibacterium*, and significantly decreased the abundance of *Turicibacter*. As one of the most common probiotics, *Lactobcillus* has long been considered to be beneficial to body health [45]. *Ileibacterium*, as a pathogenic bacterium, is positively correlated with serum lipid levels and metabolic disorders [46], so the relationship between increased *Ileibacterium* and glucose metabolism disorder needs to be further investigated via a single-strain experiment. *Turicibacter* is considered an important genus for the generation of SCFAs [47]. We found that the abundance of *Turicibacter* was significantly decreased, which might positively associate with glucose metabolism disorder [48]. Therefore, we speculated that SB removal promoted glucose metabolism disorder, which might correlate with gut microbiota dysbiosis, and a subsequent verification could be performed using a single-strain experiment. Finally, we also found that SCFA levels of the RB group were insignificant in comparison with the R group, which indicated that SCFA failed to change regardless of the presence or removal of SB. These results showed that continuous intake of SB for 10 weeks positively regulated the gut microbiota, but the special effect of SB removal on the gut microbiota needs to be further investigated using a single-strain experiment.

In summary, we investigated the effect of SB intake with different concentrations and at different stages on host health and found that SB intake for 10 weeks was safe for host metabolism and positively regulated the gut microbiota, which was also supported by an FMT experiment. However, SB intake for 5 weeks and SB removal produced some adverse influences, and related mechanisms need to be further investigated via a single-strain experiment and multi-omics technologies. This work confirmed the safety of SB intake for 10 weeks and was helpful for the dietary strategy of SB.

## Data Availability

The data used to support the findings of this study can be made available by the corresponding author upon request.

## Supplementary Materials

The following supporting information can be downloaded at: https://www.mdpi.com/article/10.3390/foods12224081/s1, 16S sequencing reads of each group.
